# E-Scooter-Associated Injury Types and Injury Severity: A Systematic Review and Meta-Analysis

**DOI:** 10.3390/jcm15062154

**Published:** 2026-03-12

**Authors:** Wiebke Käckenmester, Alexander Hönning, Heinrich Bernhard Herman Voß, Cosima Prahm, Georg Osterhoff, Julia Seifert

**Affiliations:** 1 Center for Clinical Research, BG Klinikum Unfallkrankenaus Berlin gGmbH, 12683 Berlin, Germany; alexander.hoenning@ukb.de (A.H.); cosima.prahm@ukb.de (C.P.); 2 Department of Trauma and Orthopedic Surgery, BG Klinikum Unfallkrankenaus Berlin gGmbH, 12683 Berlin, Germany; heinrichbernhardherman.voss@ukb.de (H.B.H.V.); georg.osterhoff@ukb.de (G.O.); julia.seifert@ukb.de (J.S.); 3 Department of Hand, Replantation, and Microsurgery, Charité—Universitätsmedizin Berlin, 10117 Berlin, Germany

**Keywords:** e-scooters, electric scooters, injury types, injury patterns, injury severity

## Abstract

**Background**: In the past ten years, the number of publications on injuries associated with electric scooters (e-scooters) has been increasing continuously. The aim of this systematic review and meta-analysis was to synthesize the original study results on injury types, injury severity, clinical care, accident mechanisms, risk factors, and patient characteristics associated with e-scooter accidents. **Methods**: The literature search was conducted in PubMed, EMBASE and Medline. We included quantitative clinical studies published between 07/2019 and 07/2024 that report e-scooter-associated injuries in patients who presented to an emergency department. Variables that were reported as proportions (e.g., frequency of extremity fractures) were summarized using a proportional meta-analysis. Parameters on a continuous scale were combined using a meta-analysis of the arithmetic means. **Results**: Among 524 unique records, 149 articles met the inclusion criteria, and 68 were eligible for quantitative analyses. Most e-scooter patients sustained injuries to the head and face with a pooled frequency of 42.1% (95% CI 38.7–45.4). Injuries of the upper extremities were estimated at 40.1% of patients (95% CI 35.8–44.4). Fractures of the extremities occurred with a pooled frequency of 25.7% (95% CI 22.5–28.9). An estimated proportion of 2.3% (95% CI 1.6–3.0) sustained severe traumatic brain injuries. Determined by the Injury Severity Score (ISS), 2.8% (95% CI 1.5–4.1) of the e-scooter patients were severely injured (ISS ≥ 16). **Conclusions**: Injuries to the head and face as well as the upper extremities are the most common causes for emergency department visits following e-scooter accidents. One in four patients presented with extremity fractures. Severe injuries, however, affect less than three percent of e-scooter patients.

## 1. Introduction

Electric scooters (e-scooters) increasingly contribute to road traffic injuries worldwide, especially in urban areas. Consequently, many countries introduced new regulations and laws aiming at primary prevention, such as speed limits, no-parking zones, or mandatory helmet use. A few large cities have even prohibited sharing companies from offering rental e-scooters. One reason behind these measures were reports of severe injuries sustained in e-scooter accidents. However, the incidence and patterns of severe injuries have not yet been thoroughly investigated. Although several clinical studies have been published on e-scooter-associated injuries, most were monocentric, and the ones that were collected in the initial years of e-scooter use rely on relatively small samples. However, the number of publications on e-scooter-associated injuries has been increasing steadily over the past 10 years. Furthermore, we found eight systematic reviews that have been published on this topic [1,2,3,4,5,6,7,8]. However, most of them report data that have been published earlier than 2021 [1,4,7,8]. In most countries, e-scooter legislations was introduced between 2018 and 2021. Therefore, relatively few clinical studies have been published within the early years of e-scooter use and they usually rely on small samples. More recent systematic reviews included more studies but only report specific injury types [2,6] (e.g., maxillofacial injuries). Another more recent review compared electric bicycle injuries to e-scooter-related injuries [3]. The most recent meta-analysis, including 25 observational studies, was conducted with a similar approach to our work but focused on the incidence of upper-limb injuries and head trauma [5]. Moreover, the authors did not perform a quantitative analysis of injury severity due to insufficient data. Given the growing number of publications but still lacking a meta-analysis of the severity of e-scooter injuries, we reasoned that a comprehensive review and meta-analysis may significantly advance the current state of knowledge in this field and provide a robust prior probability for clinical management.

## 2. Materials and Methods

The primary objective was to characterize the types of injuries and the severity of injuries sustained in e-scooter accidents. Secondary objectives were patient characteristics, clinical treatment, risk factors, accident mechanisms, and the relative frequency of e-scooter patients who presented to an emergency department.

### 2.1. Search Strategy

The protocol for this review was prospectively registered in the international prospective register of systematic reviews (PROSPERO, CRD42024574738) on 30 November 2024. The described subgroup analysis comparing German to non-German samples was not performed due to an insufficient number of studies. Otherwise, the registered protocol was followed. The review was conducted in accordance with the Preferred Reporting Items for Systematic reviews and Meta-Analyses (PRISMA) [9] see Supplementary Materials. We searched the electronic bibliographic databases PubMed, EMBASE, and Medline via OVID. The results were filtered for publication date (in the last 5 years) and article language (English or German). The PubMed search strategy described in Table 1 was last searched on 24 July 2024. Additionally, the reference lists of included articles were searched for further publications. We included publications starting from July 2019, as small electric vehicles, including e-scooters, were first permitted to participate in German public road traffic at this time, followed thereafter by the majority of European countries introducing similar regulations.

### 2.2. Selection Criteria

First, we identified and removed duplicate records. Next, titles and abstracts were screened for eligibility, followed by a full-text review of the articles. To be included, the articles had to (1) examine the injuries of patients who presented to an emergency department following e-scooter accidents; (2) report e-scooter-specific data; and (3) have been published between July 2019 and July 2024 in a peer-reviewed journal. Articles were excluded if they: (1) did not report original data (e.g., comments, reviews, meta-analyses); (2) did not analyze clinical data (e.g., questionnaire studies, biomechanical analyses); (3) did not describe electric scooters (e.g., pedelecs, hoverboards, nonelectric scooters); (4) reported only single cases (case studies); or (5) described the study in an abstract only (e.g., conference abstracts). We did not exclude articles based on sample characteristics such as age, gender, nationality or any other demographic aspect. E-scooter riders, as well as other road users who were injured in an e-scooter accident (e.g., pedestrians, bicycle riders), were included.

### 2.3. Data Extraction

Two reviewers independently undertook the screening of articles, data extraction, and risk of bias assessment. For each included study, the following data were extracted: year of publication, first author, data collection period, study type, patient population, sample size, country and city, participant’s age, participant’s sex, helmet use, alcohol use, substance use, time of the accident (time of day, month, season), accident mechanism (e.g., fall, collision), injury severity, road users (e.g., e-scooter riders, pedestrians), injury type, clinical care, duration of inpatient treatment, mortality, and proportion of e-scooter patients among all presentations to an emergency department.

### 2.4. Risk of Bias

The bias potential of the included studies was evaluated using the Risk of Bias Tool by Hoy et al. [10], which was developed for assessing prevalence studies. Two reviewers assessed the risk of bias for each included publication across 10 domains. The resulting sum values corresponded to low, moderate or high risk of bias. The robvis tool [11] version 1.0 was utilized to visualize the individual risk of bias assessment for each study, as well as to generate a risk of bias summary table.

### 2.5. Statistical Analyses

If the variables were presented as proportions (e.g., frequency of injury types), the data from the individual studies were analyzed using a proportional meta-analysis. If the parameters were continuous (e.g., age), the particular values were combined using a meta-analysis of the arithmetic means. A random effects model and REML (restricted maximum likelihood) estimator were used for the pooled analysis. We conducted a sensitivity analysis to compare injury type and injury severity between studies with a low risk of bias and those that included all studies, in order to evaluate the effect of bias on study estimates. The statistical analysis was performed using STATA version 16.1 [12].

The heterogeneity of the results between individual studies was assessed via forest plots and I^2^ statistics. The following values were used as cutoffs for classifying heterogeneity [13]: I^2^ > 25% to ≤50%: low heterogeneity; I^2^ > 50% to ≤75%: moderate heterogeneity; and I^2^ > 75%: high heterogeneity.

The following body regions and injury types were reported in a sufficient number of studies for quantitative analyses: head/face, upper extremities, lower extremities, abdomen/thorax/torso, back/spine, severe traumatic brain injuries, and extremity fractures. Reports of intracranial hemorrhage and skull fractures were categorized as severe traumatic brain injuries. All fractures of the extremities were summarized under extremity fractures.

## 3. Results

**Figure 1:**
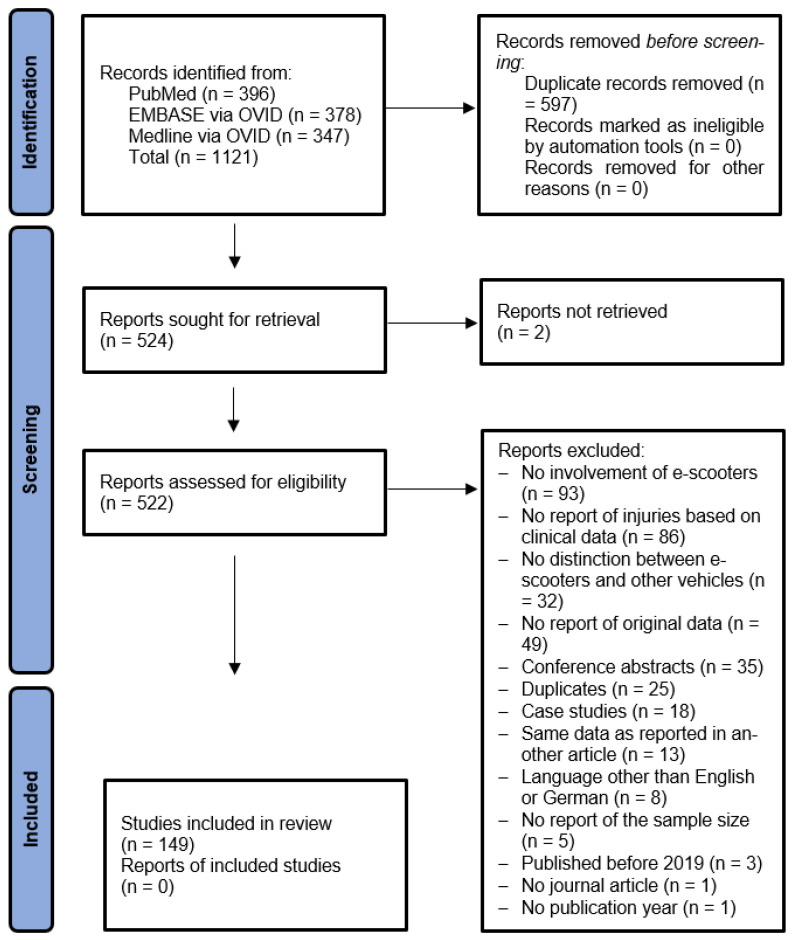
PRISMA flow chart illustrating the identification and selection process.

The automated search resulted in 524 unique records (see PRISMA flow chart, Figure 1). Following the manual screening of titles, abstracts and full texts, 149 articles met the inclusion criteria. 

Studies were eligible for quantitative analyses if the reported target population included patients with any type of e-scooter-associated injury. If the reported samples were restricted to certain injury types (e.g., maxillofacial injuries), the studies had to be excluded from the quantitative analyses. A total of 68 studies were eligible for quantitative analyses. The majority were retrospective cohort studies (76%, *n* = 52), followed by prospective cohort studies (16%, *n* = 11) and studies based on registry entries (7.3%, *n* = 5) (see Table 2).

### 3.1. Risk of Bias

**Figure 2:**
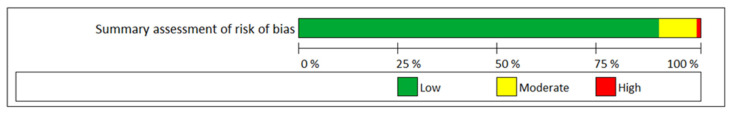
Risk of bias of the included studies.

The risk of bias assessment revealed that 132 of the included 149 studies (89%) had a low risk of bias, 15 studies (10%) had a moderate risk of bias, and only two studies (1%) had a high risk of bias (see Figure 2). The individual risk of bias assessment for each study across the ten domains is presented in Figure A15.

Among the 68 studies included in the quantitative analyses, the proportions of studies with moderate risk of bias (*n* = 5; 7%) and high risk of bias (*n* = 0) were comparable. Accordingly, a sensitivity analysis yielded almost identical estimates for injury type and injury severity when comparing the analysis based on studies with a low risk of bias to the analysis that included all studies.

### 3.2. Injury Types

**Figure A1:**
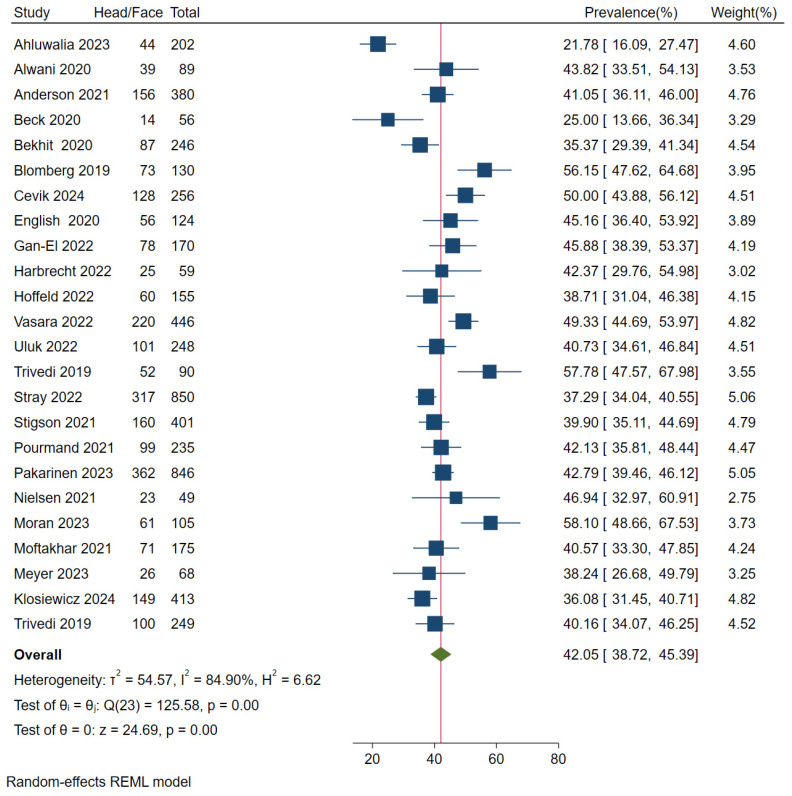
Proportion of patients with head or face injuries. The blue square represents the study-specific prevalence, the horizontal line represent its 95% confidence interval. The green diamond represents the pooled prevalence across all studies, also indicated by a red vertical line. Included articles: Ahluwalia 2023 [24], Alwani 2020 [68], Anderson 2021 [58], Beck 2020 [69], Bekhit 2020 [70], Blomberg 2019 [77], Cevik 2024 [15], English 2020 [71], Gan-El 2022 [44], Harbrecht 2022 [46], Hoffeld 2022 [48], Vasara 2022 [55], Uluk 2022 [54], Trivedi 2019 [80], Stray 2022 [53], Stigson 2021 [66], Pourmand 2021 [64], Pakarinen 2023 [37], Nielsen 2021 [63], Moran 2023 [36], Moftakhar 2021 [62], Meyer 2023 [34], Klosiewicz 2024 [19], Trivedi 2019 [81].

**Figure A2:**
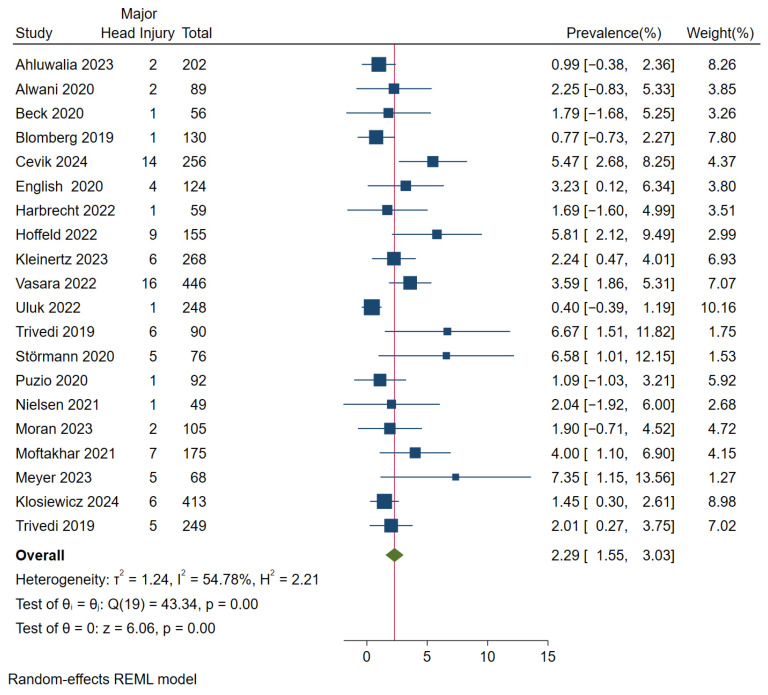
Proportion of patients with severe traumatic brain injuries. The blue square represents the study-specific prevalence, the horizontal line represent its 95% confidence interval. The green diamond represents the pooled prevalence across all studies, also indicated by a red vertical line. Included articles: Ahluwalia 2023 [24], Alwani 2020 [68], Beck 2020 [69], Blomberg 2019 [77], Cevik 2024 [15], English 2020 [71], Harbrecht 2022 [46], Hoffeld 2022 [48], Kleinertz 2023 [31], Vasara 2022 [55], Uluk 2022 [54], Trivedi 2019 [80], Störmann 2020 [74], Puzio 2020 [73], Nielsen 2021 [63], Moran 2023 [36], Moftakhar 2021 [62], Meyer 2023 [34], Klosiewicz 2024 [19], Trivedi 2019 [81].

Based on 24 publications, the frequency of patients with head/face injuries varied between 21.8% and 58.1%. The pooled proportion of head/face injuries was 42.1% (95% CI 38.7–45.4). Indicating high heterogeneity, the variation between studies resulted in I^2^ = 84.9% (see Figure A1). Severe head injuries occurred with a pooled proportion of 2.3% (95% CI 1.6–3.0) of the patients. Among the 20 available studies, the frequency ranged from 0.4% to 7.4%, with moderate heterogeneity (I^2^ = 54.8%) (see Figure A2).

**Figure A3:**
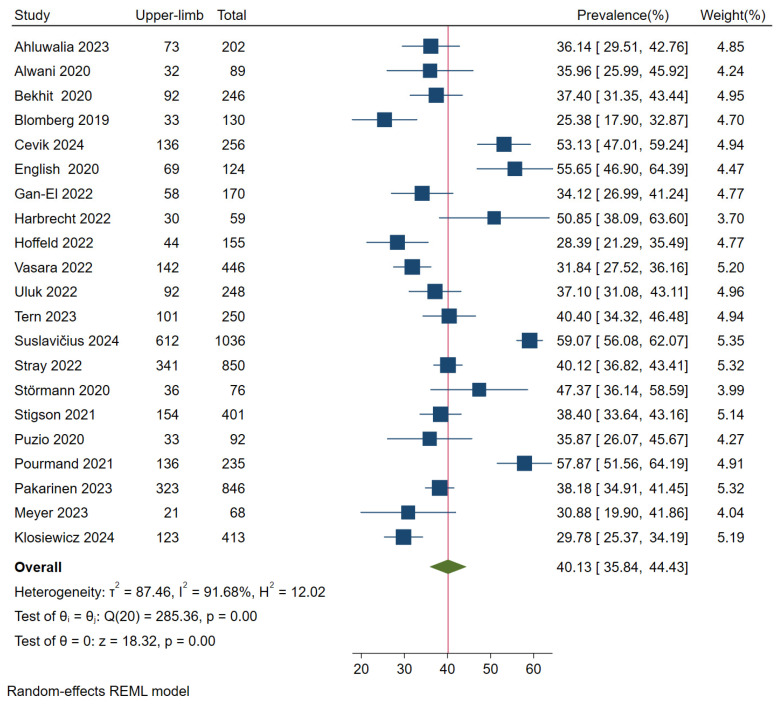
Proportion of patients with upper-limb injuries. The blue square represents the study-specific prevalence, the horizontal line represent its 95% confidence interval. The green diamond represents the pooled prevalence across all studies, also indicated by a red vertical line. Included articles: Ahluwalia 2023 [24], Alwani 2020 [68], Bekhit 2020 [70], Blomberg 2019 [77], Cevik 2024 [15], English 2020 [71], Gan-El 2022 [44], Harbrecht 2022 [46], Hoffeld 2022 [48], Vasara 2022 [55], Uluk 2022 [54], Tern 2023 [40], Suslavičius 2024 [22], Stray 2022 [53], Störmann 2020 [74], Stigson 2021 [66], Puzio 2020 [73], Pourmand 2021 [64], Pakarinen 2023 [37], Meyer 2023 [34], Klosiewicz 2024 [19].

**Figure A4:**
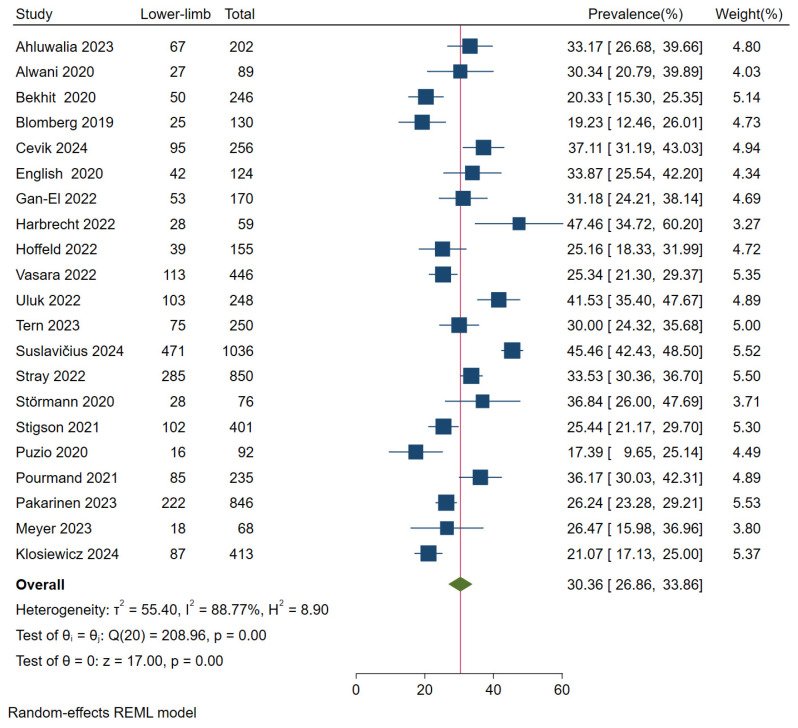
Proportion of patients with lower-limb injuries. The blue square represents the study-specific prevalence, the horizontal line represent its 95% confidence interval. The green diamond represents the pooled prevalence across all studies, also indicated by a red vertical line. Included articles: Ahluwalia 2023 [24], Alwani 2020 [68], Bekhit 2020 [70], Blomberg 2019 [77], Cevik 2024 [15], English 2020 [71], Gan-El 2022 [44], Harbrecht 2022 [46], Hoffeld 2022 [48], Vasara 2022 [55], Uluk 2022 [54], Tern 2023 [40], Suslavičius 2024 [22], Stray 2022 [53], Störmann 2020 [74], Stigson 2021 [66], Puzio 2020 [73], Pourmand 2021 [64], Pakarinen 2023 [37], Meyer 2023 [34], Klosiewicz 2024 [19].

Twenty-one studies reported how many patients suffered injuries to the upper extremities and lower extremities. Injuries to the upper extremities were reported in 25.4% to 59.1% of the samples. In the random-effects model, a pooled estimate of 40.1% (95% CI 35.8–44.4) was determined for upper-extremity injuries (see Figure A3). Injuries to the lower extremities were documented less frequently, with a pooled proportion of 30.4% (95% CI 26.9–33.9). The proportions of the individual studies ranged from 17.4% to 47.5%. The large variability between the original studies resulted in an I^2^ of approximately 90% for both upper and lower extremities (see Figure A4).

**Figure A5:**
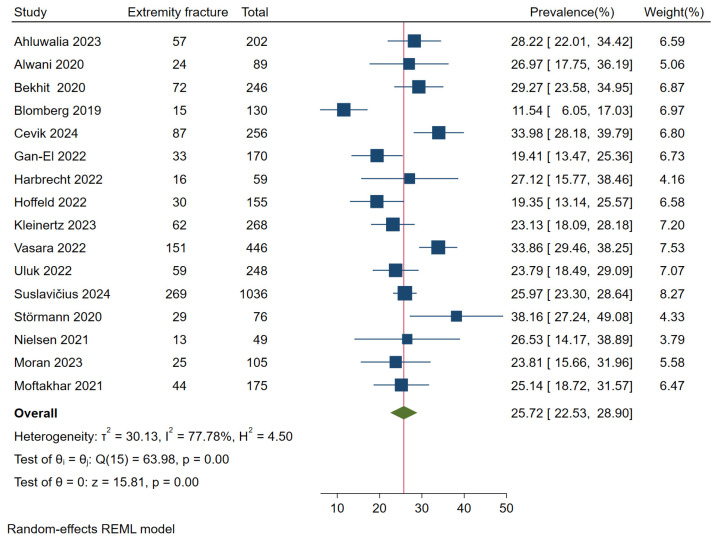
Proportion of patients with extremity fractures. The blue square represents the study-specific prevalence, the horizontal line represent its 95% confidence interval. The green diamond represents the pooled prevalence across all studies, also indicated by a red vertical line. Included articles: Ahluwalia 2023 [24], Alwani 2020 [68], Bekhit 2020 [70], Blomberg 2019 [77], Cevik 2024 [15], Gan-El 2022 [44], Harbrecht 2022 [46], Hoffeld 2022 [48], Kleinertz 2023 [31], Vasara 2022 [55], Uluk 2022 [54], Suslavičius 2024 [22], Störmann 2020 [74], Nielsen 2021 [63], Moran 2023 [36], Moftakhar 2021 [62].

Extremity fractures were documented in 16 studies, with a minimum of 11.5% and a maximum of 34.0%. Across all available studies, a pooled estimate of 25.7% (95% CI 22.5–28.9) was determined for extremity fractures. The heterogeneity was high, with an I^2^ of 77.8% (see Figure A5).

**Figure A6:**
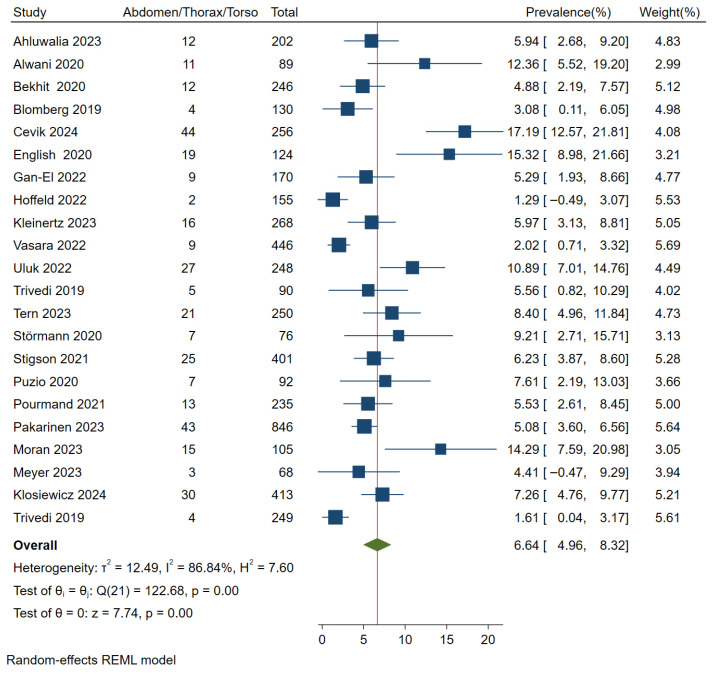
Proportion of patients with injuries to the abdomen or thorax. The blue square represents the study-specific prevalence, the horizontal line represent its 95% confidence interval. The green diamond represents the pooled prevalence across all studies, also indicated by a red vertical line. Included articles: Ahluwalia 2023 [24], Alwani 2020 [68], Bekhit 2020 [70], Blomberg 2019 [77], Cevik 2024 [15], English 2020 [71], Gan-El 2022 [44], Hoffeld 2022 [48], Kleinertz 2023 [31], Vasara 2022 [55], Uluk 2022 [54], Trivedi 2019 [80], Tern 2023 [40], Störmann 2020 [74], Stigson 2021 [66], Puzio 2020 [73], Pourmand 2021 [64], Pakarinen 2023 [37], Moran 2023 [36], Meyer 2023 [34], Klosiewicz 2024 [19], Trivedi 2019 [81].

**Figure A7:**
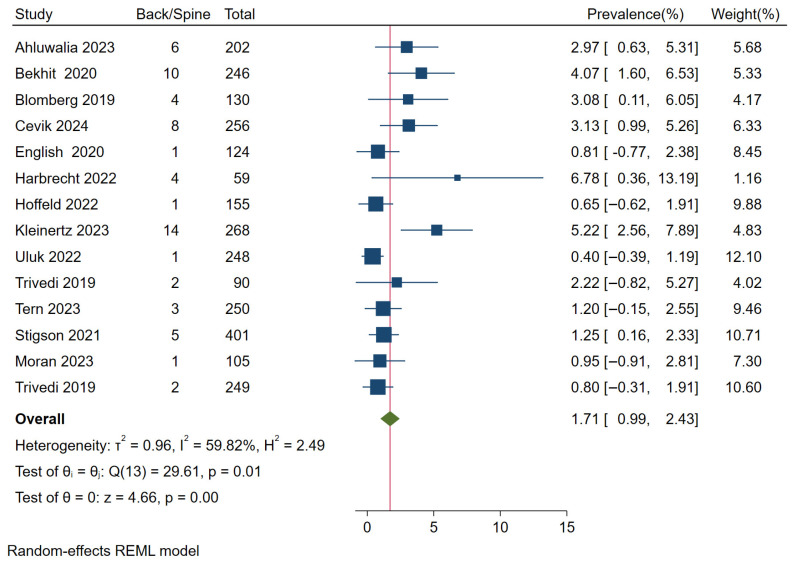
Proportion of patients with injuries to the back or spine. The blue square represents the study-specific prevalence, the horizontal line represent its 95% confidence interval. The green diamond represents the pooled prevalence across all studies, also indicated by a red vertical line. Included articles: Ahluwalia 2023 [24], Bekhit 2020 [70], Blomberg 2019 [77], Cevik 2024 [15], English 2020 [71], Harbrecht 2022 [46], Hoffeld 2022 [48], Kleinertz 2023 [31], Uluk 2022 [54], Trivedi 2019 [80], Tern 2023 [40], Stigson 2021 [66], Moran 2023 [36], Trivedi 2019 [81].

The categories abdomen/thorax/torso and back/spine were much less frequently affected by e-scooter-associated injuries. In the abdomen/thorax/torso region, the proportions ranged from 1.3% to 17.2% in 22 studies (see Figure A6). The pooled proportion was estimated at 6.6% (95% CI 5.0–8.3), with high heterogeneity (I^2^ = 86.8%). The pooled proportion of patients with injuries to the back or spine was only 1.7% (95% CI 1.0–2.4). The frequency varied between 0.4% and 6.8%. The heterogeneity for the back/spine region was considered moderate (I^2^ = 59.8%, see Figure A7).

A summary of the pooled proportions of affected body regions and injury types can be found in Table 3.

### 3.3. Severity of Injuries

**Figure 3:**
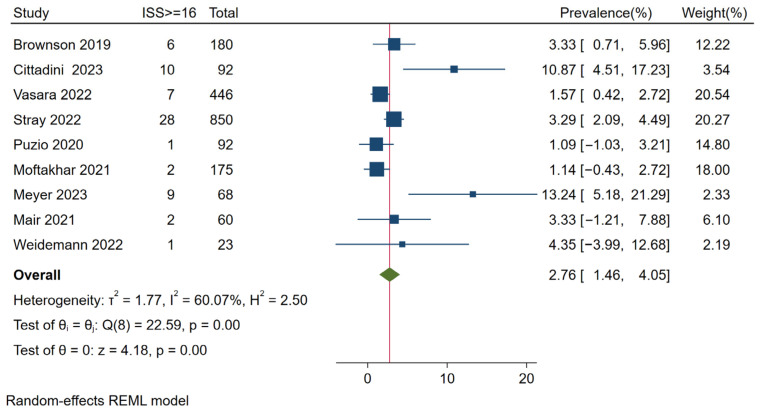
Proportion of patients with ISS ≥ 16. The blue square represents the study-specific prevalence, the horizontal line represent its 95% confidence interval. The green diamond represents the pooled prevalence across all studies, also indicated by a red vertical line. Included articles: Brownson 2019 [78], Cittadini [28], Vasara [55], Stray 2022 [53], Puzio [73], Moftakhar 2021 [62], Meyer 2023 [34], Mair 2021 [61], Weidemann 2022 [56].

The originally planned analysis of the mean/median Injury Severity Score (ISS) could not be conducted because of a lack of the respective ISS parameters reported in the original studies. Nine original studies presented ISS results in a categorized format, primarily ISS < 16 vs. ISS ≥ 16. Therefore, we focused the analysis on patients with an ISS ≥ 16, referring to the proportion of seriously injured patients [82]. The proportion of seriously injured patients ranged from 1.1% to 13.2%. Notably, seven of nine studies reported that fewer than 5% of the patients were seriously injured, whereas the other two studies reported proportions of 10.9% and 13.2%. The pooled proportion of patients with an ISS ≥ 16 was 2.8% (95% CI 1.5–4.1). The heterogeneity was moderate (I^2^ = 60.0%) (see Figure 3).

### 3.4. Clinical Treatment

**Figure A8:**
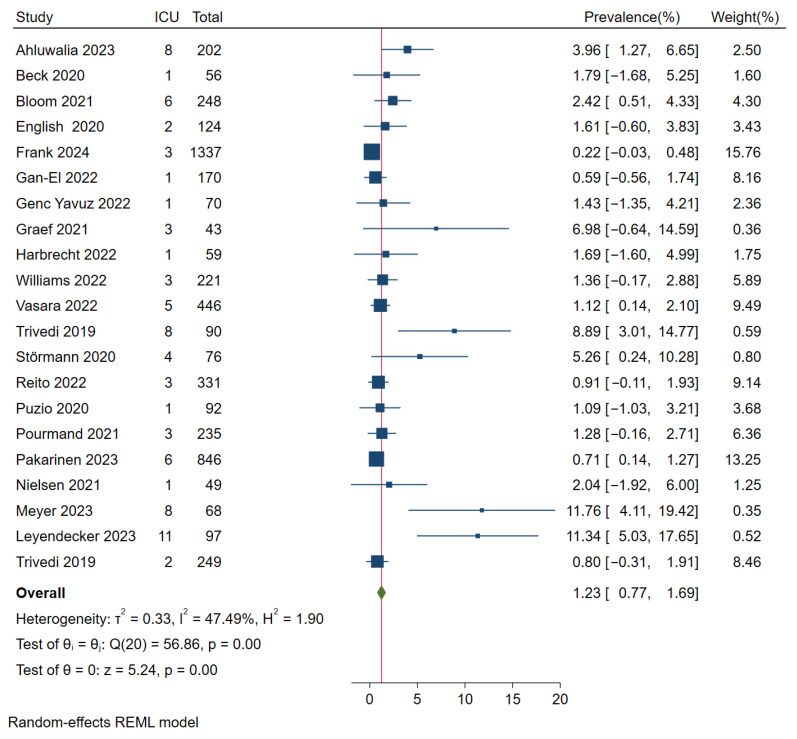
Proportion of patients who were treated in an intensive care unit (ICU). The blue square represents the study-specific prevalence, the horizontal line represent its 95% confidence interval. The green diamond represents the pooled prevalence across all studies, also indicated by a red vertical line. Included articles: Ahluwalia 2023 [24], Beck 2020 [69], Bloom 2021 [59], English 2020 [71], Frank 2024 [16], Gan-El 2022 [44], Genc Yavuz 2022 [45], Graef 2021 [60], Harbrecht 2022 [46], Williams 2022 [57], Vasara 2022 [55], Trivedi 2019 [80], Störmann 2020 [74], Reito 2022 [51], Puzio 2020 [73], Pourmand 2021 [64], Nielsen 2021 [63], Meyer 2023 [34], Leyendecker 2023 [32], Trivedi 2019 [81].

In 49 studies, the proportion of hospitalized patients ranged from 2% to 70.6%. The pooled proportion of patients who received inpatient treatment was 18.6% (95% CI 15.0–22.1). The heterogeneity was high (I^2^ = 98.0%). The pooled proportion of patients treated in an intensive care unit was 1.2% (95% CI 0.8–1.7) (see Figure A8).

**Figure 4:**
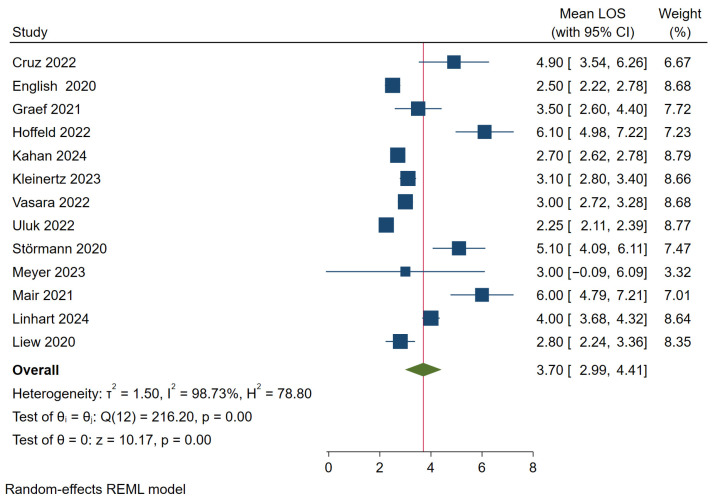
Proportion of hospitalized patients. The blue square represents the study-specific arithmetic mean, the horizontal line represent its 95% confidence interval. The green diamond represents the pooled arithmetic mean estimate across all studies, also indicated by a red vertical line. Included articles: Cruz 2022 [43], Englisch 2020 [71], Graef 2021 [60], Hoffeld 2022 [48], Kahan 2024 [17], Kleinertz 2023 [31], Vasara 2022 [55], Uluk 2022 [54], Störmann 2020 [74], Meyer 2023 [34], Mair 2021 [61], Linhart 2024 [20], Liew 2020 [72].

The average length of hospital stay ranged between 2.2 days and 6.1 days. The pooled estimate was 3.7 days (95% CI 3.0–4.4). The wide range between studies was reflected in an I^2^ of 98.7% (see Figure 4).

### 3.5. Patient Characteristics

**Figure A9:**
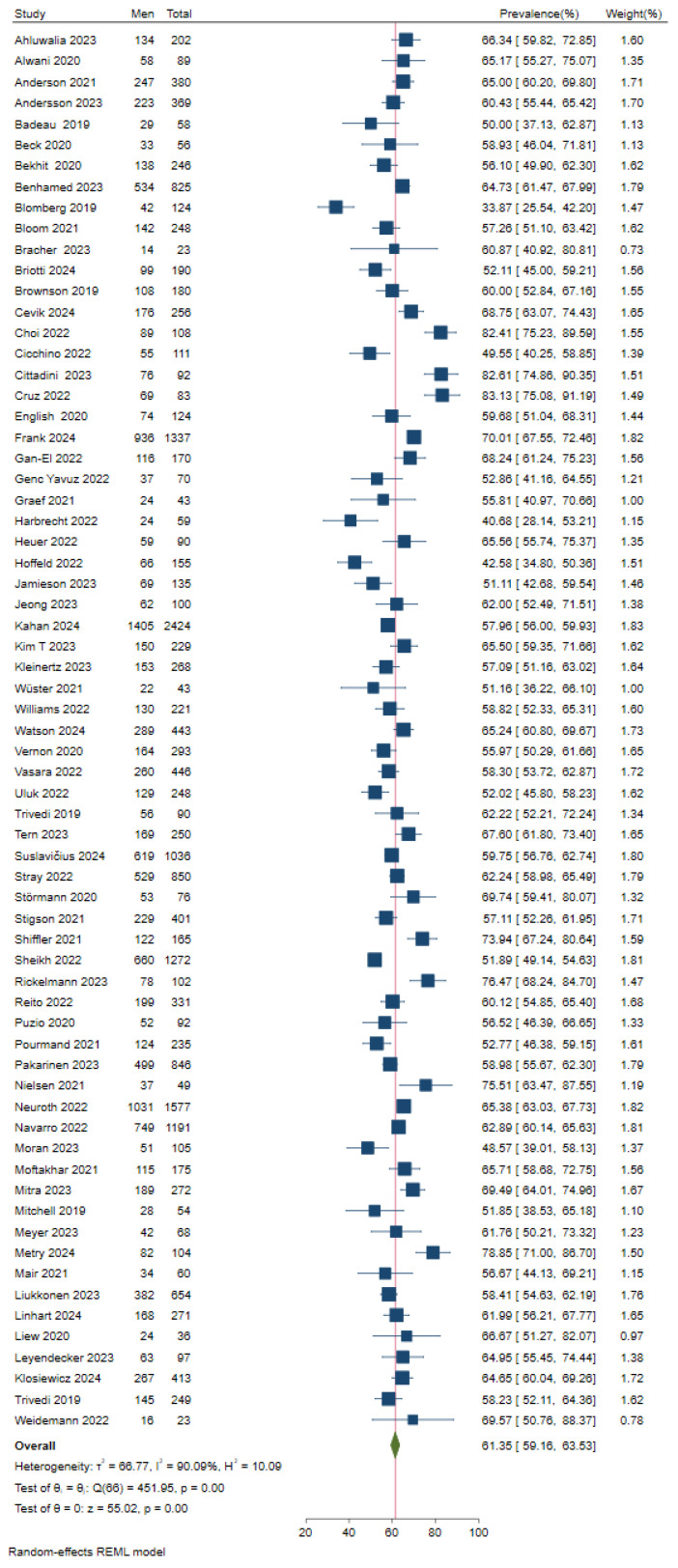
Proportion of male patients. The blue square represents the study-specific prevalence, the horizontal line represent its 95% confidence interval. The green diamond represents the pooled prevalence across all studies, also indicated by a red vertical line. Included articles: Ahluwalia 2023 [24], Alwani 2020 [68], Anderson 2021 [58], Andersson 2023 [25], Badeau 2019 [76], Beck 2020 [69], Bekhit 2020 [70], Benhamed 2023 [26], Blomberg 2019 [77], Bloom 2021 [59], Bracher 2023 [27], Briotti 2024 [14], Brownson 2019 [78], Choi 2022 [41], Cevik 2024 [15], Cicchino 2022 [42], Cittadini 2023 [28], Cruz 2022 [43], English 2020 [71], Frank 2024 [16], Gan-El 2022 [44], Genc Yavuz 2022 [45], Graef 2021 [60], Harbrecht 2022 [46], Heuer 2022 [47], Hoffeld 2022 [48], Jamieson 2023 [29], Jeong 2022 [30], Kahan 2024 [17], Kim T 2023 [18], Kleinertz 2023 [31], Wüster 2021 [67], Williams 2022 [57], Watson 2024 [23], Vernon 2020 [75], Vasara 2022 [55], Uluk 2022 [54], Trivedi 2019 [80], Tern 2023 [40], Suslavičius 2024 [22], Stray 2022 [53], Störmann 2020 [74], Stigson 2021 [66], Shiffler 2021 [65], Sheikh 2022 [52], Rickelmann 2023 [38], Reito 2022 [51], Puzio 2020 [73], Pourmand 2021 [64], Pakarinen 2023 [37], Nielsen 2021 [63], Neuroth 2022 [50], Navarro 2022 [49], Moran 2023 [36], Moftakhar 2021 [62], Mitra 2023 [35], Mitchell 2019 [79], Meyer 2023 [34], Metry 2024 [21], Mair 2020 [61], Liukkonen 2023 [33], Linhart 2024 [20], Liew 2020 [72], Leyendecker 2023 [32], Klosiewicz 2024 [19], Trivedi 2019 [81], Weidemann 2022 [56].

**Figure A10:**
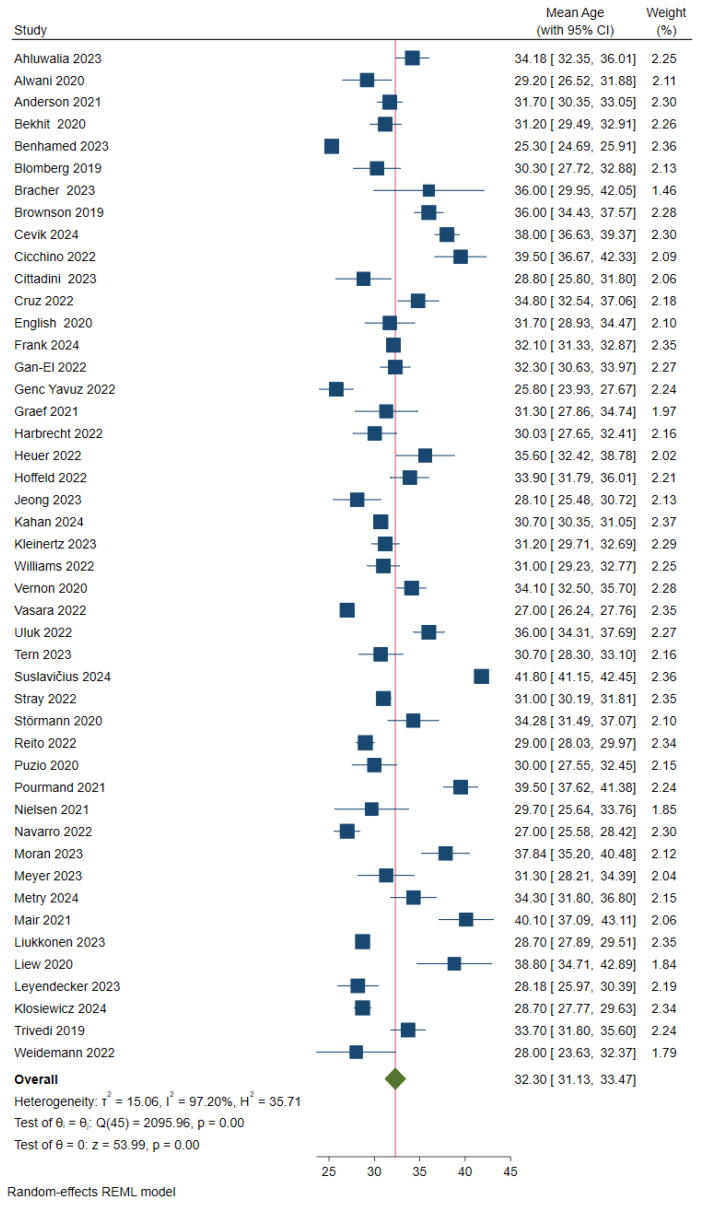
Mean age of the included patients. The blue square represents the study-specific arithmetic mean, the horizontal line represent its 95% confidence interval. The green diamond represents the pooled arithmetic mean estimate across all studies, also indicated by a red vertical line. Included articles: Ahluwalia 2023 [24], Alwani 2020 [68], Anderson 2021 [58], Bekhit 2020 [70], Benhamed 2023 [26], Blomberg 2019 [77], Bracher 2023 [27], Brownson 2019 [78], Choi 2022 [41], Cevik 2024 [15], Cicchino 2022 [42], Cittadini 2023 [28], Cruz 2022 [43], English 2020 [71], Frank 2024 [16], Gan-El 2022 [44], Genc Yavuz 2022 [45], Graef 2021 [60], Harbrecht 2022 [46], Heuer 2022 [47], Hoffeld 2022 [48], Jeong 2022 [30], Kahan 2024 [17], Kleinertz 2023 [31], Williams 2022 [57], Vernon 2020 [75], Vasara 2022 [55], Uluk 2022 [54], Tern 2023 [40], Suslavičius 2024 [22], Stray 2022 [53], Störmann 2020 [74], Reito 2022 [51], Puzio 2020 [73], Pourmand 2021 [64], Nielsen 2021 [63], Navarro 2022 [49], Moran 2023 [36], Meyer 2023 [34], Mair 2020 [61], Liukkonen 2023 [33], Liew 2020 [72], Leyendecker 2023 [32], Klosiewicz 2024 [19], Trivedi 2019 [81], Weidemann 2022 [56].

On the basis of 67 studies, the pooled proportion of male patients was estimated at 61.4% (95% CI 59.2–63.5). The individual proportions of male patients varied between 33.9% and 83.1%, resulting in high heterogeneity (I^2^ = 90.1%, see Figure A9). The mean age reported in 46 studies ranged from 25.3 to 41.8 years. The pooled estimate was 32.3 years (95% CI 31.1–33.5). The heterogeneity between the individual study results was high (I^2^ = 97.2%, see Figure A10). Approximately half of the analyzed studies were conducted in European countries (35 of 68, 51.5%). Nineteen studies (27.9%) were conducted in the USA or Canada, ten (14.7%) in Australia or New Zealand, three (4.4%) in South Korea, and one (1.5%) in Singapore (see Table 2).

### 3.6. Wearing a Helmet and Alcohol Intoxication

**Figure A11:**
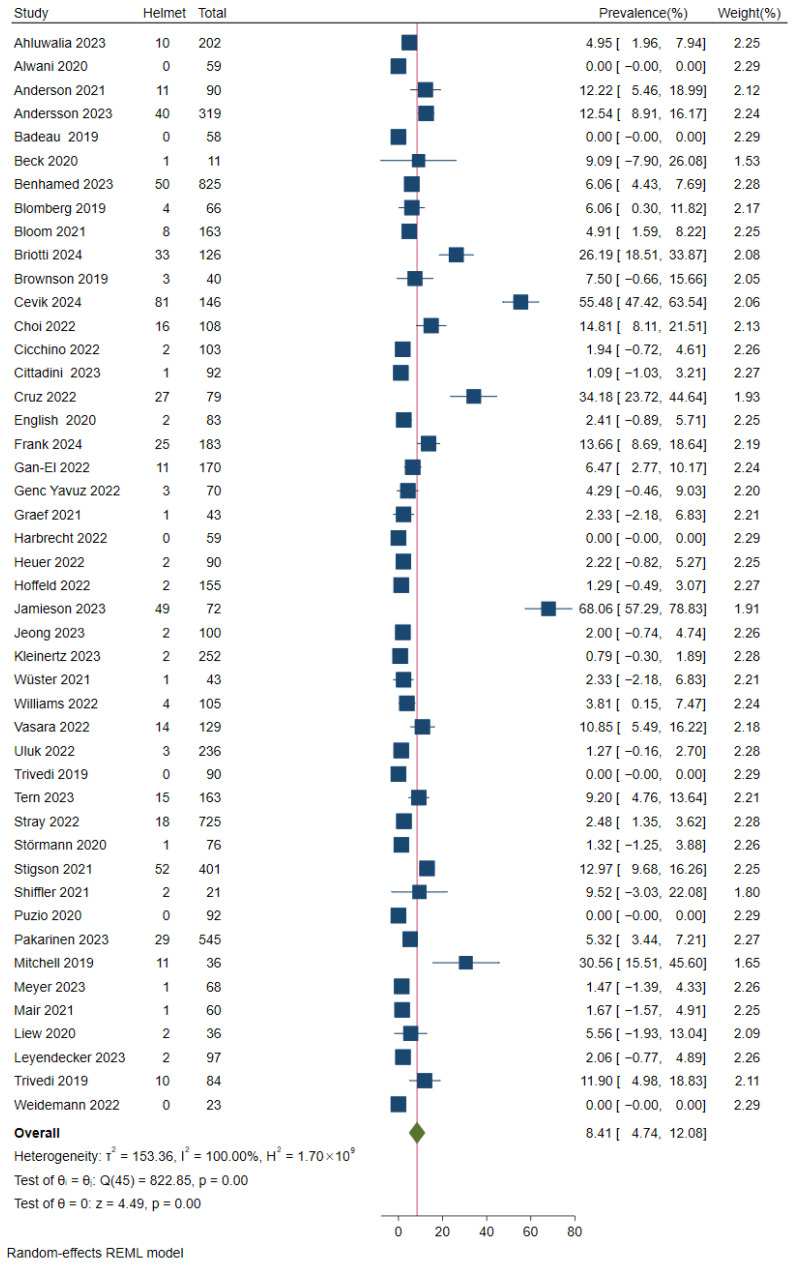
Proportion of patients who wore a helmet at the time of the accident. The blue square represents the study-specific prevalence, the horizontal line represent its 95% confidence interval. The green diamond represents the pooled prevalence across all studies, also indicated by a red vertical line. Included articles: Ahluwalia 2023 [24], Alwani 2020 [68], Anderson 2021 [58], Andersson 2023 [25], Badeau 2019 [76], Beck 2020 [69], Benhamed 2023 [26], Blomberg 2019 [77], Bloom 2021 [59], Briotti 2024 [14], Brownson 2019 [78], Cevik 2024 [15], Choi 2022 [41], Cicchino 2022 [42], Cittadini 2023 [28], Cruz 2022 [43], English 2020 [71], Frank 2024 [16], Gan-El 2022 [44], Genc Yavuz 2022 [45], Graef 2021 [60], Harbrecht 2022 [46], Heuer 2022 [47], Hoffeld 2022 [48], Jamieson 2023 [29], Jeong 2022 [30], Kleinertz 2023 [31], Wüster 2021 [67], Williams 2022 [57], Vasara 2022 [55], Uluk 2022 [54], Trivedi 2019 [80], Tern 2023 [40], Stray 2022 [53], Störmann 2020 [74], Stigson 2021 [66], Shiffler 2021 [65], Puzio 2020 [73], Pakarinen 2023 [37], Mitchell 2019 [79], Meyer 2023 [34], Mair 2020 [61], Liew 2020 [72], Leyendecker 2023 [32], Trivedi 2019 [81], Weidemann 2022 [56].

The proportion of patients wearing a helmet was reported in 46 studies. However, for the majority of the patients (in some studies, even more than 90%), no information was available on whether the patient was wearing a helmet. A large variation between the individual studies was evident in the forest plot. In 41 of 46 studies, fewer than one in seven patients wore a helmet. The other five studies reported significantly higher proportions, and in two studies, the reported proportion of patients wearing helmets even exceeded 50% of the sample. Consequently, the heterogeneity reached a maximum of I^2^ = 100%. Owing to the very heterogeneous individual results, the estimated proportion of 8.4% (95% CI 4.7–12.1) must be interpreted with caution (see Figure A11).

**Figure A12:**
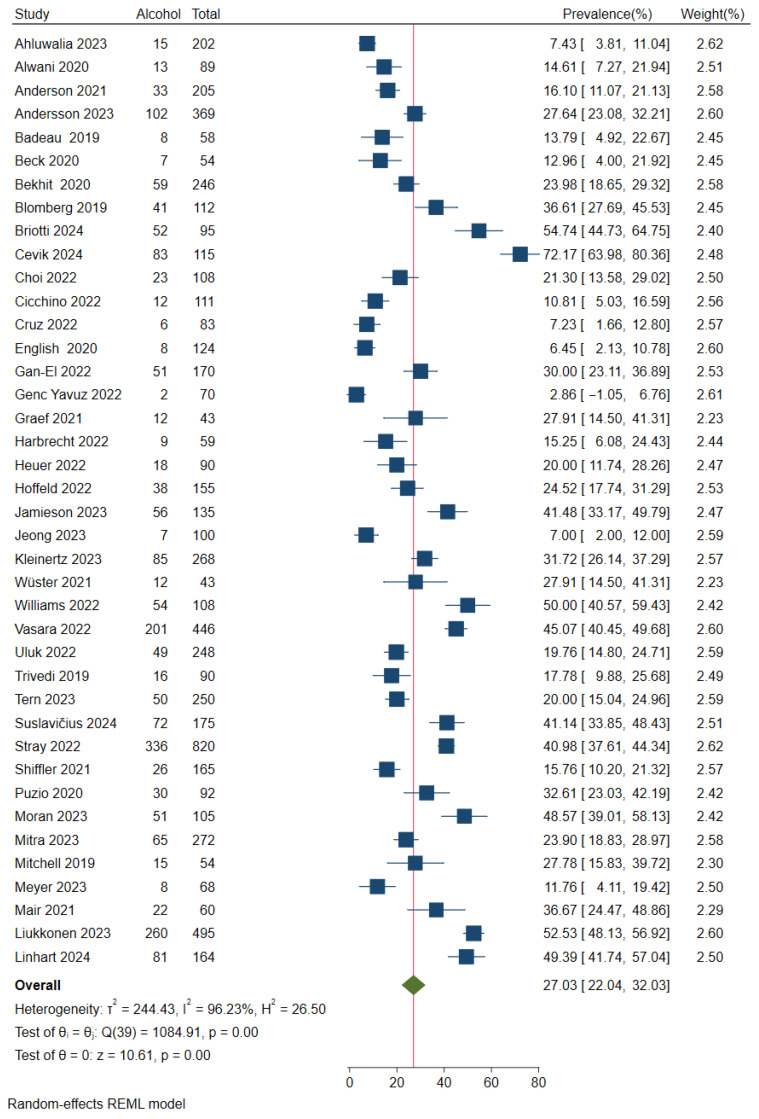
Proportion of patients who were reportedly intoxicated with alcohol. The blue square represents the study-specific prevalence, the horizontal line represent its 95% confidence interval. The green diamond represents the pooled prevalence across all studies, also indicated by a red vertical line. Included articles: Ahluwalia 2023 [24], Alwani 2020 [68], Anderson 2021 [58], Andersson 2023 [25], Badeau 2019 [76], Beck 2020 [69], Bekhit 2020 [70], Blomberg 2019 [77], Briotti 2024 [14], Cevik 2024 [15], Choi 2022 [41], Cicchino 2022 [42], Cruz 2022 [43], English 2020 [71], Gan-El 2022 [44], Genc Yavuz 2022 [45], Graef 2021 [60], Harbrecht 2022 [46], Heuer 2022 [47], Hoffeld 2022 [48], Jamieson 2023 [29], Jeong 2022 [30], Kleinertz 2023 [31], Wüster 2021 [67], Williams 2022 [57], Vasara 2022 [55], Uluk 2022 [54], Trivedi 2019 [80], Tern 2023 [40], Suslavičius 2024 [22], Stray 2022 [53], Shiffler 2021 [65], Puzio 2020 [73], Moran 2023 [36], Mitra 2023 [35], Mitchell 2019 [79], Meyer 2023 [34], Mair 2020 [61], Liukkonen 2023 [33], Linhart 2024 [20].

The proportion of patients intoxicated with alcohol was reported in 40 publications. Similar to the helmet wearing rate, for approximately 80% of patients, no information was available on alcohol consumption. The variance between studies was high, ranging from 2.9% to 72.2%. Owing to the high heterogeneity (I^2^ = 96.2%), the pooled estimate of 27.0% (95% CI 22.0–32.0) is of limited significance when determining how frequently e-scooter patients have been injured under the influence of alcohol (see Figure A12).

### 3.7. Accident Mechanisms

**Figure A13:**
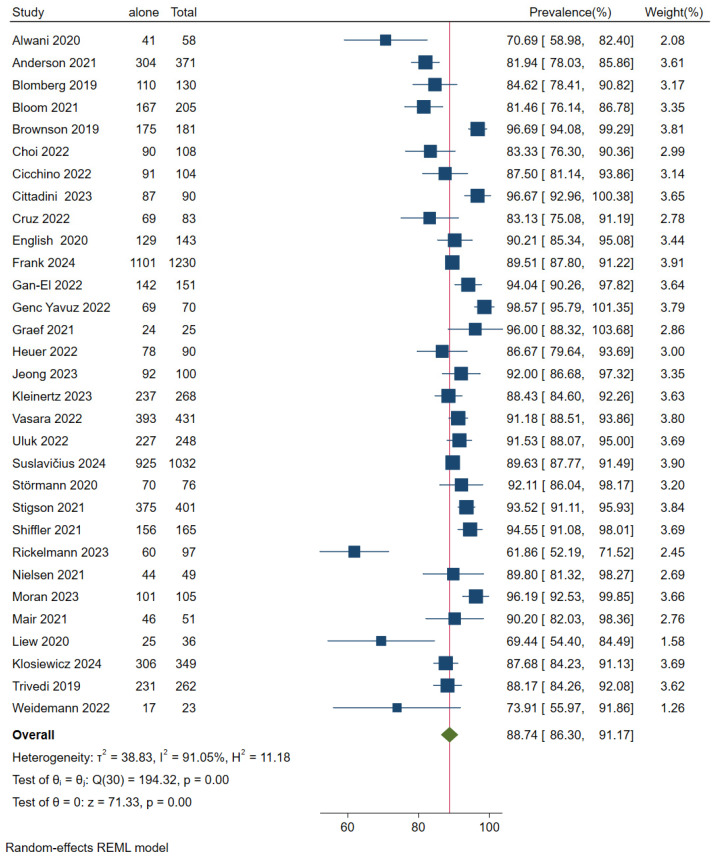
Proportion of patients injured in a single-vehicle accident. The blue square represents the study-specific prevalence, the horizontal line represent its 95% confidence interval. The green diamond represents the pooled prevalence across all studies, also indicated by a red vertical line. Included articles: Alwani 2020 [68], Anderson 2021 [58], Blomberg 2019 [77], Bloom 2021 [59], Brownson 2019 [78], Choi 2022 [41], Cicchino 2022 [42], Cittadini 2023 [28], Cruz 2022 [43], English 2020 [71], Frank 2024 [16], Gan-El 2022 [44], Genc Yavuz 2022 [45], Graef 2021 [60], Heuer 2022 [47], Jeong 2022 [30], Kleinertz 2023 [31], Vasara 2022 [55], Uluk 2022 [54], Suslavičius 2024 [22], Störmann 2020 [74], Stigson 2021 [66], Shiffler 2021 [65], Rickelmann 2023 [38], Nielsen 2021 [63], Moran 2023 [36], Mair 2020 [61], Liew 2020 [72], Klosiewicz 2024 [19], Trivedi 2019 [81], Weidemann 2022 [56].

**Figure A14:**
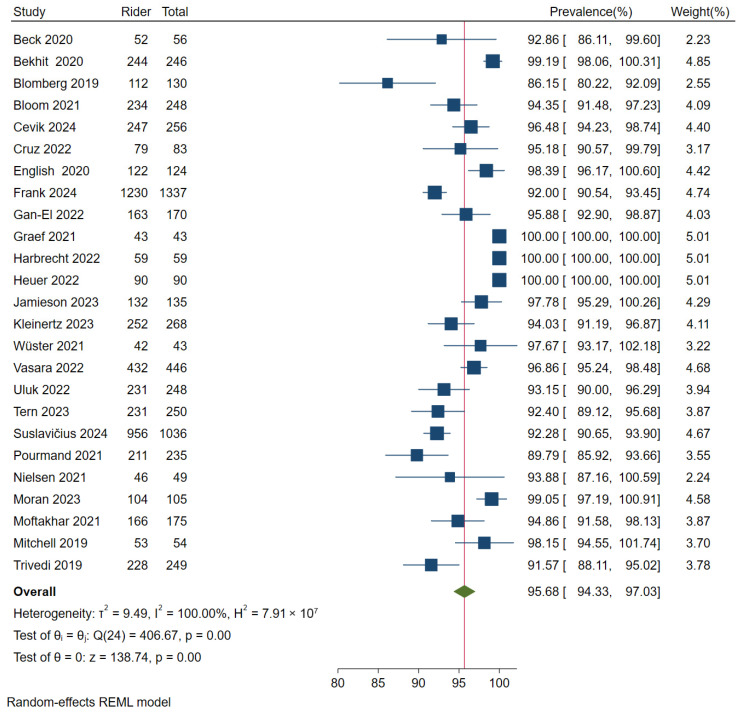
Proportion of e-scooter riders. The blue square represents the study-specific prevalence, the horizontal line represent its 95% confidence interval. The green diamond represents the pooled prevalence across all studies, also indicated by a red vertical line. Included articles: Beck 2020 [69], Bekhit 2020 [70], Blomberg 2019 [77], Bloom 2021 [59], Cevik 2024 [15], Cruz 2022 [43], English 2020 [71], Frank 2024 [16], Gan-El 2022 [44], Graef 2021 [60], Harbrecht 2022 [46], Heuer 2022 [47], Jamieson 2023 [29], Kleinertz 2023 [31], Wüster 2021 [68], Vasara 2022 [55], Uluk 2022 [54], Tern 2023 [40], Suslavičius 2024 [22], Pourmand 2021 [64], Nielsen 2021 [63], Moran 2023 [36], Moftakhar 2021 [62], Mitchell 2019 [79], Trivedi 2019 [81].

**Figure A15:**
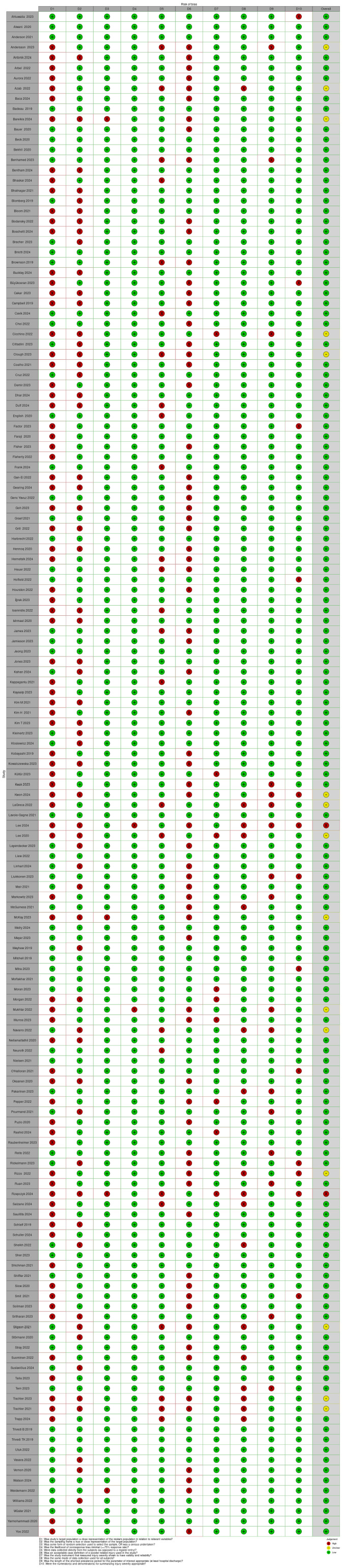
Risk of bias assessment of each included study. Included articles: Azab 2022 [1], Ahluwalia 2023 [24], Alwani 2020 [68], Anderson 2021 [58], Andersson 2023 [25], Baca 2024 [83], Badeau 2019 [76], Beck 2020 [69], Bekhit 2020 [70], Benhamed 2023 [26], Blomberg 2019 [77], Bloom 2021 [59], Bracher 2023 [27], Briotti 2024 [43], Brownson 2019 [78], Cevik 2024 [15], Choi 2022 [41], Cicchino 2022 [42], Cittadini 2023 [28], Clough 2023 [83], Cruz 2022 [43], English 2020 [71], Frank 2024 [16], Gan-El 2022 [44], Genc Yavuz 2022 [45], Goh 2023 [4], Graef 2021 [60], Harbrecht 2022 [46], Heuer 2022 [47], Hoffeld 2022 [48], Jamieson 2023 [29], Jeong 2022 [30], Kahan 2024 [17], Kim SJ 2023 [2], Kim T 2023 [18], Kleinertz 2023 [31], Klosiewicz 2024 [19], Leyendecker 2023 [32], Liew 2020 [72], Linhart 2024 [20], Liukkonen 2023 [33], Mair 2020 [61], Metry 2024 [21], Meyer 2023 [34], Mitchell 2019 [79], Mitra 2023 [35], Moftakhar 2021 [62], Moran 2023 [36], Navarro 2022 [49], Neuroth 2022 [50], Nielsen 2021 [63], Niemann 2023 [3], Pakarinen 2023 [37], Pourmand 2021 [64], Puzio 2020 [73], Reito 2022 [51], Rickelmann 2023 [38], Sheikh 2022 [52], Sher 2023 [39], Shiffler 2021 [65], Singh 2022 [8], Spota 2024 [5], Stassen 2024 [6], Stigson 2021 [66], Störmann 2020 [74], Stray 2022 [53], Suslavičius 2024 [22], Tern 2023 [40], Toofany 2021 [7], Trivedi 2019 [80], Trivedi 2019 [81], Uluk 2022 [54], Vasara 2022 [55], Vernon 2020 [75], Wüster 2021 [67], Williams 2022 [57], Watson 2024 [23], Weidemann 2022 [56], Yarmohammadi 2020 [90], Yoo 2022 [91].

Thirty-one studies provided information on whether the accident was a single-vehicle accident or whether other road users were involved. The majority of at least 61.9% up to 98.6% were involved in single-vehicle accidents. Across all studies, the pooled estimate was 88.7% (95% CI 86.3–91.2). With an I^2^ of 91.1%, the heterogeneity was high (see Figure A13). In 86.2% to 100% of the reported cases, the accident victims were e-scooter riders. The pooled proportion of e-scooter riders among all patients was estimated at 95.7% (95% CI 94.3–97.0), with high heterogeneity (I^2^ = 100%) (see Figure A14). The remaining patients presenting at the emergency room were almost exclusively pedestrians.

The majority of accidents occurred during spring, summer, and autumn as well as on weekends and at night. However, owing to very heterogeneous documentation, a quantitative analysis of the season, day of the week, and time of day was not possible.

### 3.8. Frequency of E-Scooter Injuries

In 2020, a study from three London hospitals reported that 1.6% (83 out of 5177) of referrals to the orthopedic department were associated with e-scooter accidents [43]. In a single-center study from Turkey covering 2021 to 2022, the proportion of referrals due to fractures caused by e-scooter accidents among all referrals to the emergency orthopedic department was 0.8% (56 out of 6881) [83]. In a Berlin hospital in 2019, 43 (0.5%) of the 9366 patients who presented to the emergency department were affected by e-scooter accidents [67]. In a dataset from 2018 of patients from two hospitals in Indiana, USA, 0.3% (92 out of 742) of all emergency room visits were caused by e-scooter accidents [68]. However, as the two-month study period was during the fall, the authors assume that this percentage was underestimated. An evaluation of the Swedish STRADA database, which documents injuries from traffic accidents, revealed that in the summer months of 2019, 14% of all emergency room visits associated with traffic accidents were related to e-scooters [25]. With 15% of all emergency room visits in the summer months of 2019 and 2020, very similar ratios were reported in Calgary [52]. A quantitative analysis of the relative frequency of e-scooter injuries was not possible due to a lack of sufficient data.

## 4. Discussion

E-scooter-associated injuries are an ongoing subject of public debate and clinical research. The aim of this systematic review and meta-analysis was to synthesize the published evidence on injury patterns, injury severity, clinical treatment, patient characteristics, risk factors, accident mechanisms, and the relative frequency of e-scooter patients who presented to an emergency department.

### 4.1. Injury Types

E-scooter patients who present to an emergency department are most commonly diagnosed with injuries to the head and face as well as the upper extremities. Injuries to the lower extremities were less frequently observed. The abdomen and thorax are rarely affected by e-scooter accidents, and the back and spine are even less affected. These findings correspond with the results of previously published systematic reviews that reported most e-scooter-related injuries as involving the upper extremities as well as the head and face [5,7]. It has been assumed that injuries to the upper extremities are often caused by extending the arms during a fall. Injuries to the face and head, on the other hand, occur when the arm’s extension reflexes cannot prevent the head from hitting an object or the ground. Although the lower extremities are affected less frequently, almost every third patient was diagnosed with injuries to the legs and feet.

It needs to be noted, that a high degree of variability in the proportions of specific injury types was observed across most entities, indicated by an I^2^ greater than 75% for head/face injuries, upper- and lower-extremity injuries, extremity fractures, and abdomen/thorax/torso injuries. This large variation probably originates, among other factors, from varying infrastructures, legislations, and healthcare systems in the countries where the studies were conducted. Therefore, the point estimates for the observed proportions should always be interpreted in conjunction with the confidence interval.

One in four patients presented with extremity fractures. Thus, extremity fractures, mainly fractures of the upper extremities, are a common and relevant consequence of e-scooter-related accidents. Other common e-scooter-associated fractures affect the facial bones. The proportion of patients with facial bone fractures could not be estimated quantitatively because of a lack of available data. However, maxillofacial fractures are reported frequently and should be considered a relevant consequence of e-scooter accidents.

Severe traumatic brain injuries occurred in approximately two percent of all patients. Notably, serious brain injuries might be more common in e-scooter accidents than bicycle accidents [84,85]. Possible explanations are e-scooter-specific accident mechanisms and risk factors such as riding under the influence of alcohol and without a helmet. Alcohol reduces the reaction times and reflexes in the event of a fall. Therefore, it has been assumed that alcohol consumption results in fewer limb injuries and more head injuries [31,86]. Furthermore, e-scooter riders are less likely to wear a helmet than cyclists are, which poses further risks of serious brain injuries [3].

### 4.2. Injury Severity

The Injury Severity Score (ISS) was the most commonly reported measure of injury severity. With an ISS of at least 16, the proportion of seriously injured patients was less than three percent. A more detailed analysis of the ISS parameters was not possible due to a lack of consistent data. However, the relatively small proportion of severely injured patients indicates that most e-scooter patients who present to the emergency departments have minor or moderate injuries.

### 4.3. Clinical Care

Among all patients who presented to an emergency department, less than twenty percent were admitted to the ward. However, the study results were highly heterogeneous, indicating that regional or international differences might have caused varying admission rates (e.g., bed capacities, hospital densities, or the availability of outpatient services). Among all e-scooter patients registered in the emergency department, only one percent was treated in an intensive care unit.

The average length of hospital stay across all available studies was almost four days. However, the variation between studies was considerably high. The length of hospital stay might also be influenced by health care parameters such as bed capacities, billing systems, or health insurance.

### 4.4. Patient Characteristics

With sixty-one percent, the gender of e-scooter patients was predominantly male. The observed gender ratio corresponds with an overall greater proportion of male e-scooter riders [87] and a greater proportion of males who sustain injuries in accidents [88]. E-scooter patients who presented to the emergency departments were, on average, 32 years old. Research on patients who sustained injuries in bicycle accidents suggests that e-scooter patients are significantly younger than injured cyclists [3].

With regard to both patient characteristics, gender and age, the between study-heterogeneity was very high, with an I^2^ greater than 90%. This indicates substantial variability in age distribution and gender proportions across the individual studies.

### 4.5. Accident Mechanisms

With ninety-six percent of all patients, the accident victims were almost exclusively e-scooter riders. Other injured road users were pedestrians, cyclists, and passengers (co-riders). Pedestrians accounted for the largest proportion of injured non-riders. However, they made up for less than five percent of all e-scooter-injured patients. Due to unclear sample selection in some studies, the number of injured non-riders might be slightly underestimated, which most likely contributed significantly to the observed maximum heterogeneity, with an I^2^ of 100%. Nevertheless, riders apparently have a much higher risk of being injured in e-scooter accidents than pedestrians or cyclists. Among the injured e-scooter riders, nearly ninety percent suffered a single-vehicle accident (i.e., an accident not involving other road users). The most common accident mechanisms were falls, followed by collisions with stationary objects. The high rate of single-vehicle accidents and the proportion of falls as the most common accident mechanism were in accordance with findings of previous systematic reviews [5,7].

### 4.6. Risk Factors

Approximately one-quarter of the e-scooter patients were reportedly intoxicated with alcohol. However, the results must be interpreted with caution because of a high rate of missing values. Furthermore, most available data were based on self-reports or subjective external assessments, and blood alcohol levels were reported in only a minority of publications. The proportion of alcohol-intoxicated e-scooter patients might be lower than the results of retrospective clinical studies suggest. For comparison, fifteen percent of e-scooter accident victims had increased blood alcohol levels according to German police reports [89]. Nevertheless, police reports confirm that e-scooter riders were significantly more often intoxicated with alcohol than injured cyclists. In the future, prospective clinical studies are necessary to investigate the incidence of alcohol intoxication as well as correlations between enhanced blood alcohol levels and the frequency of severe injuries.

Another proposed risk factor for severe brain injuries has been the low rate of helmet use among e-scooter riders. On average, less than ten percent of the injured e-scooter riders reportedly wore a helmet at the time of the accident. However, an average of eighty percent missing values limits the significance of the results. Furthermore, we assume the high heterogeneity was partly attributable to helmet legislations. In countries that require helmet wearing, more than half of the injured e-scooter riders wore helmets [15,29]. Thus, mandatory helmet use increases the probability of helmet wearing and as a result might reduce the incidence of severe brain injuries. Moreover, riding bans at night-time have been suggested to prevent severe injuries caused by poor visibility at night and riding under the influence of alcohol. In the future, prospective clinical studies are necessary to investigate the incidence of helmet use as well as correlations between helmet use and the frequency of severe head injuries.

### 4.7. Frequency of E-Scooter Injuries

In two publications, the proportion of e-scooter patients was reported in relation to the total number of patients who presented to the emergency department [67,68]. The proportion of e-scooter patients was 0.3% and 0.5%, respectively. However, the data were collected in the early years of e-scooter legalization (2018–2022). Given the increasing number of e-scooter users, the relative frequency of e-scooter patients might have increased in recent years. The proportion of e-scooter patients among the total number of patients treated in the same facility is a useful indicator of the impact that e-scooters have on health care services. In addition to the total number of emergency department visits, subgroups of patients with relevant injuries (e.g., upper extremity fractures) might serve as a reference group to describe the relative frequency of e-scooter patients in future studies.

### 4.8. Limitations

The large variation across the original studies in reporting the injury types made it difficult to group injury patterns. Some injuries were described using a variety of symptoms, leaving only a few options for creating categories (e.g., traumatic brain injuries). In most studies that reported the ISS, the results were described only briefly, or the reported statistical parameters were not sufficiently consistent to allow for merging of the original study results. Therefore, we were not able to analyze average ISS scores as originally planned. The results concerning risk factors for severe injuries, such as alcohol intoxication or riding without a helmet, were of limited significance due to high rates of missing values and subjective methods. To overcome these limitations, the influence of risk factors on e-scooter injuries should be studied in prospective designs.

A limitation when interpreting the results was the high heterogeneity observed in most proportion analyses. Partly, the large variance between the individual study results might be attributed to differences between regions or countries with varying infrastructures, legislations, and health care systems. Furthermore, the documentation in the original studies did not permit an analysis of the relationship between e-scooter type and the frequency and severity of injuries. Therefore, we were unable to examine the influence of maximum speed, the weight of the e-scooter, motor power, or wheel size.

## 5. Conclusions

Extremity fractures and severe traumatic brain injuries are among the most relevant consequences of e-scooter accidents. However, the majority of patients treated in the emergency departments sustained minor injuries and only less than three percent were classified as severely injured. Nevertheless, the increasing use of e-scooters leads to more accidental injuries, additional visits to the emergency departments, and hospital admissions. More prospective studies are needed to identify risk factors that increase the likelihood of severe e-scooter injuries.

## Figures and Tables

**Table 1:** PubMed search strategy.

Steps	Query	Results
1.	(e-scooter *) OR (electr * scooter *) OR (escooter *) OR (motorized scooter *) OR (powered scooter *)	431
2.	(Wounds and injuries [MesH]) OR (injur *) OR (trauma *) OR (fracture) OR (bone) OR (laceration) OR (burn) OR (wound)	793,483
3.	(Surgical Procedures, Operative [MesH]) OR (Emergency Service, Hospital [MesH]) OR (Hospitalization [MeSH]) OR (operat *) OR (surg *) OR (admission) OR (emergency) OR (hospital *)	3,524,033
4.	(Accidents [MesH]) OR (accident *) OR (collision) OR (crash) OR (fall)	88,129
5.	1. AND 2.	314
6.	1. AND 3.	317
7.	1. AND 4.	284
8.	5. OR 6. OR 7.	381

* term for truncation used in the PubMed search syntax to search for word stems

**Table 2:** Characteristics of the 68 studies included in the quantitative analyses.

First Author	Year	Study Period	Study Design (Number of Centers)	Study Place	Sample Size
Briotti [14]	2024	2023–2024	retrospective monocentric	Australia, Broome	190
Cevik [15]	2024	2022–2023	retrospective monocentric	Australia, Melbourne	256
Frank [16]	2024	2018–2021	retrospective monocentric	Austria, Vienna	1337
Kahan [17]	2024	2020–2023	retrospective monocentric	USA, Denver	2424
Kim [18]	2024	2018–2022	retrospective monocentric	South Korea, Konyang	229
Klosiewicz [19]	2024	2020–2023	retrospective multicentric (*n* = 2)	Poland	413
Linhart [20]	2024	2019–2022	retrospective monocentric	Germany, Munich	271
Metry [21]	2024	2017–2022	retrospective monocentric	England, London	104
Suslavičius [22]	2024	2018–2021	retrospective monocentric	Lithuania, Vilnius	1036
Watson [23]	2024	2022–2023	retrospective monocentric	Australia, Brisbane	443
Ahluwalia [24]	2023	2020–2020	retrospective monocentric	England, London	202
Andersson [25]	2023	2019–2020	register study (*n* unknown)	Sweden, Stockholm	369
Benhamed [26]	2023	2019–2019	register study (*n* unknown)	France, Rhone	825
Bracher [27]	2023	2019–2021	retrospective monocentric	Switzerland, Bern	23
Cittadini [28]	2023	2019–2022	retrospective monocentric	Italy, Rome	92
Jamieson [29]	2023	2021–2022	retrospective monocentric	Australia, Hobart	135
Jeong [30]	2023	2020–2020	retrospective monocentric	South Korea, Incheon	100
Kleinertz [31]	2023	2019–2021	retrospective monocentric	Germany, Hamburg	268
Leyendecker [32]	2023	2021	retrospective monocentric	Germany, Cologne	97
Liukkonen [33]	2023	2019–2022	retrospective monocentric	Finland, Tampere	654
Meyer [34]	2023	2019–2020	prospective monocentric	Germany, Essen	68
Mitra [35]	2023	2017–2022	retrospective multicentric (*n* = 2)	Australia, Melbourne	272
Moran [36]	2023	2021	prospective multicentric (*n* = 2)	Australia, Darwin	105
Pakarinen [37]	2023	2021–2022	retrospective multicentric (*n* = 3)	Finland, Helsinki	846
Rickelmann [38]	2023	2018–2022	retrospective monocentric	USA, Norfolk	102
Sher [39]	2023	2019–2022	retrospective monocentric	USA, Tampa	292
Tern [40]	2023	2021	prospective multicentric (*n* = 20)	UK	250
Choi [41]	2022	2018–2021	retrospective monocentric	South Korea, Gwangju	108
Cicchino [42]	2022	2019	prospective monocentric	USA, Washington DC	111
Cruz [43]	2022	2020–2020	retrospective multicentric (*n* = 3)	England, London	83
Gan-El [44]	2022	2019–2020	prospective monocentric	Belgium, Brussels	170
Genc Yavuz [45]	2022	2020–2020	retrospective monocentric	Turkey, Istanbul	70
Harbrecht [46]	2022	2019–2020	prospective monocentric	Germany, Cologne	59
Heuer [47]	2022	2019–2020	retrospective monocentric	Germany, Hamburg	90
Hoffeld [48]	2022	2019–2021	retrospective monocentric	Germany, Munich	155
Navarro [49]	2022	2013–2018	register study (*n* ca. 100)	USA	1191
Neuroth [50]	2022	2015–2019	register study (*n* ca. 100)	USA	1577
Reito [51]	2022	2019–2021	retrospective monocentric	Finland, Tampere	331
Sheikh [52]	2022	2019–2020	retrospective multicentric (*n* unknown)	Canada, Calgary	1272
Stray [53]	2022	2019–2020	retrospective monocentric	Norway, Oslo	850
Uluk [54]	2022	2019	prospective multicentric (*n* = 4)	Germany, Berlin	248
Vasara [55]	2022	2021	retrospective multicentric (*n* = 3)	Finland, Helsinki	446
Weidemann [56]	2022	2020–2021	prospective monocentric	Germany, Hannover	23
Williams [57]	2022	2018–2019	retrospective multicentric (*n* = 2)	USA, St. Louis	221
Anderson [58]	2021	2018–2020	retrospective monocentric	USA, Atlanta	380
Bloom [59]	2021	2018–2018	retrospective monocentric	USA, Los Angeles	248
Graef [60]	2021	2019–2019	retrospective monocentric	Germany, Berlin	43
Mair [61]	2021	2019–2020	prospective monocentric	Germany, Munich	60
Moftakhar [62]	2021	2018–2019	retrospective multicentric (*n* = 3)	Austria, Vienna	175
Nielsen [63]	2021	2019	prospective multicentric (*n* = 2)	Denmark, Copenhagen	49
Pourmand [64]	2021	2017–2019	retrospective monocentric	USA, Washington DC	235
Shiffler [65]	2021	2017–2019	retrospective multicentric (*n* unknown)	USA, Los Angeles	165
Stigson [66]	2021	2019–2020	register study (*n* unknown)	Sweden	401
Wüster [67]	2021	2019	retrospective monocentric	Germany, Berlin	43
Alwani [68]	2020	2018–2018	retrospective multicentric (*n* unknown)	USA, Indiana	89
Beck [69]	2020	2018–2019	retrospective monocentric	Australia, Dunedin	56
Bekhit [70]	2020	2018–2019	retrospective multicentric (*n* = 5)	New Zealand, Auckland	246
English [71]	2020	2018	retrospective mul-ticentric (*n* = 2)	USA, Texas	124
Liew [72]	2020	2015–2016	retrospective monocentric	Singapore, Singapore	36
Puzio [73]	2020	2018	retrospective multicentric (*n* = 2)	USA, Indiana	92
Störmann [74]	2020	2019–2020	prospective multicentric (*n* = 2)	Germany, Frankfurt	76
Vernon [75]	2020	2018–2019	retrospective multicentric (*n* = 4)	USA, Atlanta	293
Badeau [76]	2019	2017–2018	retrospective monocentric	USA, Salt Lake City	58
Blomberg [77]	2019	2016–2019	retrospective monocentric	Denmark, Copenhagen	130
Brownson [78]	2019	2018–2019	retrospective monocentric	New Zealand, Auckland	180
Mitchell [79]	2019	2018–2019	retrospective monocentric	Australia, Brisbane	54
Trivedi [80]	2019	2018–2019	retrospective monocentric	USA, Dallas	90
Trivedi [81]	2019	2017–2018	retrospective multicentric (*n* = 2)	USA, Los Angeles	249

**Table 3:** Affected body regions and injury types—summary.

	Pooled Proportion (95% CI)	Number of Studies
Head/face	42.1% (38.7–45.4)	24
Severe traumatic brain injury	2.3% (1.6–3.0)	20
Upper extremity	40.1% (35.6–44.4)	21
Lower extremity	30.4% (26.9–33.9)	21
Extremity fractures	25.7% (22.5–28.9)	16
Abdomen/thorax/torso	6.6% (5.0–8.3)	22
Back/spine	1.7% (1.0–2.4)	14

Abbreviation: CI = Confidence Interval.

## Data Availability

The data presented in this study are available on request from the corresponding author.

## Supplementary Materials

The following supporting information can be downloaded at https://www.mdpi.com/article/10.3390/jcm15062154/s1, PRISMA 2020 Checklist.

## Abbreviations

The following abbreviations are used in this manuscript:

CI	Confidence interval
e-scooter	Electric scooter
I^2^	Measure of between-study heterogeneity
N	Number
ISS	Injury Severity Score
PRISMA	Preferred Reporting Items for Systematic reviews and Meta-Analyses
PROSPERO	International prospective register of systematic reviews
%	Percentage
